# Changes in Proximate Chemical and Mineral Compositions of Different Sex Categories of Mutton during the Dry-Curing Process

**DOI:** 10.3390/ani11113019

**Published:** 2021-10-20

**Authors:** Marina Krvavica, Jelena Đugum, Marijana Drinovac Topalović, Andrijana Kegalj, Iva Ljubičić, Miljenko Konjačić

**Affiliations:** 1 Marko Marulic Polytechnic of Knin, Krešimirova 30, 22300 Knin, Croatia; mdrinovac@veleknin.hr (M.D.T.); akegalj@veleknin.hr (A.K.); iljubicic@veleknin.hr (I.L.); 2 Ministry of Agriculture, Ul. Grada Vukovara 78, 10000 Zagreb, Croatia; jelena.dugum@mps.hr; 3 Faculty of Agriculture, University of Zagreb, Svetošimunska Cesta 25, 10000 Zagreb, Croatia; mkonjacic@agr.hr

**Keywords:** chemical composition, dalmatian dry-cured mutton, effect of sex and castration, kaštradina, mineral composition, mutton

## Abstract

**Simple Summary:**

The older raw mutton categories are considered to be of poor quality and are poorly accepted by consumers, primarily because of the toughness and intense odor. Therefore, they are most often used in the production of various meat products. In Dalmatia, mutton is mainly processed into a traditional dry-cured product called *kaštradina*. Its quality depends on the age and sex, and the *kaštradina* of the castrated and fattened rams (wethers) are the best accepted by consumers. This study examined the influence of sex and castration on the proximate chemical and mineral compositions of raw mutton and *kaštradina* as indicators of their quality. Meat is primarily a source of protein and a significant source of minerals (phosphorus, iron, zinc, potassium) in the human diet. Therefore, it is important to determine which factors influence their composition the most. The results showed that sex and castration significantly affected the salt content and the proximate chemical and mineral compositions of raw mutton and *kaštradina* that could affect the quality (nutritive and sensory) of *kaštradina.* The findings suggested that the raw mutton of wethers and ewes could be a better-quality raw material for production of *kaštradina* than could the ram mutton.

**Abstract:**

The aim of this research was to determine the effect of sex, castration, and processing on the chemical properties of mutton in the production of *kaštradina*—a traditional Dalmatian dry-cured meat product. Therefore, the carcasses of 20 ewes (E), 20 rams (R), and 20 wethers (W) of the Dalmatian pramenka breed were processed by dry-curing. On the 1st, 35th, and 60th days of processing, the samples from the scapulae were taken, then the proximate chemical, NaCl, and mineral analyses were performed, and significant differences between most of the parameters were found. Unlike W, the R samples contained significantly more proteins (*p* < 0.01), NaCl (*p* < 0.05), and potassium (*p* < 0.05) and less fat (*p* < 0.05). Furthermore, compared to the W and R categories, the E category of *kaštradina* contained significantly more calcium (*p* < 0.05). The higher contents of intramuscular fat, potassium, and calcium and lower content of NaCl could positively affect the sensory (marbling, flavor, juiciness, and tenderness) and chemical (fatty acid profile) properties of *kaštradina*. These findings suggest that the W and E raw mutton could be a better-quality raw material for production of *kaštradina* than could the R, but further research is needed for a more comprehensive picture of its quality.

## 1. Introduction

Dry-cured mutton, called *kaštradina* or *koštradina*, is an autochthonous dry-cured meat product made of mutton, which can rarely be found on the Croatian market today. It is a semi-permanent or permanent dry-cured meat product from sheep or goat meat produced by salting, smoking, drying, and maturing [1]. *Kaštradina* production is mainly based on animals culled from breeding, female and male (ewes and rams), fattened male castrates, and barren sheep. However, in Dalmatia the highest quality is considered to be *kaštradina* produced from the mutton of castrated and fattened rams (wethers) [1,2]. Furthermore, the culled animals are difficult to sell and they achieve a low market price. Therefore, they achieve the best price through the production of *kaštradina* [2].

Since 2011, *kaštradina* has been on the List of Croatian gastronomic heritage dishes [3] and it is highly appreciated, especially among Dalmatian consumers. Generally, the quality of processed meat depends on raw meat quality and production technology. The quality of carcasses and meat depends on several factors that can generally be divided into internal or genetic (species, genotype, sex, age, weight) [4,5] and external or environmental factors (breeding and feeding technology, ante- and postmortem procedures) [6,7]. All of them affect the muscle structure and the course of biochemical processes in the muscles before and after slaughter, which further affects the technological, physical-chemical, sensory, and nutritional properties of meat and meat products [8]. The effect of these factors on meat quality of different animal species and categories has been investigated so far [9] and it was found that the quality of lamb meat depends on slaughter weight and fattening, sex and castration, age, and genotype [10,11,12,13]. However, there is relatively little research on the carcass and meat quality properties of older sheep categories and the factors that affect their quality [14,15]. Except for age [5], gender also affects the meat quality. Meat obtained by castrates has a less intense odor than the meat of adult sheep and especially of entire rams [13]. Additionally, its chemical composition (especially fat and protein content) is more similar to the meat of females than the meat of entire males [13,16]. Castration, i.e., the removal of the testes, reduces testosterone production (the most important androgen hormone) and its serum levels decline over time [17,18]. Since testosterone stimulates protein synthesis and increases the capacity for muscular development and growth rate [19], castration leads to reductions in growth rate and muscular development and increases fat deposition. Castration also affects other chemical properties such as fatty and amino acid profiles [13,20].

In general, meat is a significant source of minerals, especially phosphorus, iron, and zinc, and contains significantly more potassium than sodium and less calcium. Although some authors state that their content is quite uniform concerning the animal species and anatomical position [21], the mineral composition of meat can also vary because of genetic, physiological (gender), and environmental (diet) factors [22]. The results of several studies on the effect of sex on the content of minerals in meat are inconsistent. Some authors found a significant effect of gender on the content of minerals in meat [23], while others found little or no effect [24]. However, the mineral content of processed meat, especially dry-cured meat products, is significantly large, due to the increased sodium content as a consequence of the salting process. High-sodium intake poses a risk to human health, especially the cardiovascular and renal systems [25], and it is necessary to pay attention to its daily intake. Furthermore, due to coarser muscle structure, hardness, excessive fat content, and specific (unpleasant) odor and aroma, the meat of adult sheep, especially of uncastrated rams, which is often repulsive to consumers, is not suitable for fresh use. Therefore, processing technology is an additional factor in the quality of not only *kaštradina* but of meat products in general. However, previous research on *kaštradina* and similar indigenous dried-meat products from sheep meat is mainly of gastronomic and historical natures, and only some state the product properties and processing technology of *kaštradina* and other indigenous sheep meat products [1,2,26,27].

Consequently, as a part of a research study on the technology and quality properties of *kaštradina*, this study aimed to determine the impact of gender and castration on the basic chemical and mineral composition of Dalmatian traditional dry-cured *kaštradina* processed from three sex categories (ewes, wethers, and entire rams), such as proximate chemical composition, NaCl content, and composition of macro- and microelements.

## 2. Materials and Methods

### 2.1. Production of Dalmatian Kaštradina

A total of 60 sheep carcasses of the local Dalmatian pramenka breed, bred traditionally by grazing and hay, were purchased on the Dalmatian market (from a local slaughterhouse). The carcasses came from the following sex categories: 20 ewes (E), 20 rams (R), and 20 wethers (W) castrated at least 6 months before slaughter. The slaughter was carried out in mid-December, at the end of the mating season. At the slaughter, the animals were 3 to 5 years old and the average weights of cold carcasses of the above categories were 22.92 ± 3.25 kg, 29.11 ± 3.21 kg, and 26.20 ± 3.44 kg, respectively. For the production of *kaštradina*, carcasses were cut into six parts (two whole legs with hind-shank, two blade shoulders with the fore-shank, and two rest of the halves called “*kora*”) according to the traditional Dalmatian manner: After cutting each carcass in half, the whole leg (with hind-shank) was separated from each half by making the section from the loin and paunch between the last lumbar vertebra and the first cross vertebra, and then the shoulder blade with the fore-shank was separated from the neck, chest, and ribs by a circular-elliptical incision through the natural muscle connection. The rest, the third part of the half that is called “*kora*” (crust) in Dalmatia, includes the neck, part of the ribs under the shoulder blade, chest, ribs, back, loin, and paunch [1,2]. The meat samples for analyses of chemical and micro- and macroelements’ compositions were taken from the shoulder with the fore-shank. These cut carcasses’ parts went through the following process: dry-salting by sea salt (7% per kg of meat), drying + smoking and ripening. After 10 days the meat was removed from the salt, washed, drained, and put into the smoking-drying chamber. The combination of drying and cold smoking was used for 10 days. Cold smoke produced by pyrolyzing of hardwoods was applied two times (days) over 10 days. Then the meat was moved into the drying-ripening chambers with a controlled microclimate until the end of the process. During the processing, the temperature and the RH were controlled (salting: up to +4 °C; drying + smoking: +8 to +22 °C, RH 55–85%; ripening: +12 to +18 °C, RH 65–75%). The total length of the whole process was 60 days.

### 2.2. Sampling

The meat samples were taken from the shoulders of three sex categories three times during processing, on the 1st, 35th, and 60th days of processing (10 samples per category × 3 processing times; a total of 30 samples per category), each time from other shoulders. The samples were taken by a longitudinal incision on the caudal side of the shoulder from *angulus caudalis scapulae* to *tuber olecranii* on *processus olecranii ulnae*, and a piece of meat of approximately 200 g was separated, which included the following muscles: *M. triceps* (*tricipitis*) *brachii* (*caput laterale, caput caudale et caput mediale*) and *M. tensor fasciae antebrachii*. All visible adipose and connective tissue from the samples were removed, after which they were individually vacuum-packaged, coded, frozen, and stored at −20 °C until analysis. Immediately before the chemical analysis, the frozen samples were homogenized in a commercial electric meat grinder to achieve a visually homogeneous mixture.

### 2.3. Chemical Analyses

Proximate chemical analysis and analysis of macro- and microelements were carried out on both raw and matured samples of lean *kaštradina*. Fat, protein, moisture, and ash and sodium chloride contents were determined according to methods recommended by the AOAC [28] and according to Kasap et al. [24]. Results were expressed as wt% of the sample.

Determination of the content of macro- and microelements (Ca, P, Mg, Na, K, Mn, Cu, Zn, and Fe) was performed according to Kasap et al. [24].

All the analyses were done in duplicate and the average score for each sample was used for statistical analysis.

### 2.4. Statistical Analyses

Assessment of the effect of the mutton category (gender and castration) and length of the processing on the chemical properties (proximate chemical properties, content of NaCl, composition of macro- and microelements) was performed using the software package SAS (Cary, NC, USA) [29]. Data were analyzed using GLM and MIXED statistical procedure according to the model:

Y_ijk_ = µ + S_i_ + T_j_ + M_ijk_ + e_ijkl_ (1)

where Y_ijk_ is measured property, µ is overall mean value for the property, S_i_ is effect of category (i = 1, 2, 3), T_j_ is effect of the processing length (j = 1, 2, 3), M_ijk_ is initial mass of shoulder (covariable in the model), and e_ijkl_ is unexplained effect.

## 3. Results and Discussion

### 3.1. Proximate Chemical Properties of Raw Mutton and Kaštradina

The shoulder muscles of different categories of sheep (ewes, wethers, and entire rams) in this study contained, on average, 74.81% water, 19.82% protein, 4.41% fat, and 0.99% ash. According to this study, the influence of sex and castration on the proximate chemical composition of mutton was determined (Table 1). The content of most chemical ingredients (except water) in raw mutton (first day of processing) was significantly different among the categories (ewes, wethers, and entire rams). The differences in fat content (from 2.4% in the meat of uncastrated rams and 5.08% in the meat of ewes to 7.3% in the meat of wethers) were significant among all three categories. In contrast, the protein and ash contents in the meat of ewes and rams were similar and significantly higher than the content found in the meat of wethers, although, if observed through their share in dry matter (DM), they were significantly different among all three categories. These results partially coincide with the common assumption that the meat of castrates in chemical composition, especially in fat content, is very similar to the meat of females and generally contains more fat than the meat of whole male animals [30,31,32,33]. Okeudo and Moss [34] found the highest content of fat in the meat of ewes and wethers (2.50% and 2.37%), while the content in the meat of uncastrated rams was significantly lower (1.91%). Additionally, other authors [34,35] emphasized the higher proportion of intramuscular lipids in the meat of castrated boars compared to gilts, stating that this is expected given that castration stimulates intramuscular fat accumulation [36]. Many authors reported the effect of castration vs. intact males on the content of fat and protein in meat, explaining that by removing the testes that produce males’ natural anabolic steroids, testosterone and estrogen, their production is reduced [37,38]. In particular, testosterone is associated with a positive nitrogen balance, increased proteins, and decreased fat proportion in carcass [13,39].

Further, although the differences in water content in raw mutton among the studied categories were not statistically significant, there was an evident trend of decreasing water content with increasing fat content in meat, since the Pearson correlation (Table 2) between water and fat was negative (*n* = −0.63; *p* < 0.01), which is consistent with the research of Hoffman et al. [10]. Additionally, the contents of proteins and fat per DM (P/DM; F/DM) showed that the lower the proteins’ content in raw meat, the higher the fat content, since the Pearson correlation (Table 2) between proteins and DM (*n* = 0.79; *p* < 0.001) was higher than that between fat and DM (*n* = 0.63; *p* < 0.01). Investigating the production of traditional dry-cured mutton (in the Balkans region called “*stelja*”), Dumić [40] stated that the proximate chemical composition of raw mutton (*M. sartorius*) consisted of 71.33% to 72.74% water, 21.56% to 21.88% protein, 4.33% to 5.23% fat, and 1.07% to 1.14% ash, which is quite similar to the results of this study. In addition, females’ meat was also found to contain more fat than entire ram meat (similar to the results of Okeudo and Moss [41]). Consequently, a decrease in protein and ash content in castrated meat was reported, consistent with the results of other authors [41,42,43].

Changes in the proximate chemical composition of the processed mutton in the production of *kaštradina* were primarily the result of dehydration and concentration of chemical components in the muscles, since the reduction of water content after 35 days and 60 days of processing were about 26% and 36%, respectively. The influence of sex and castration on the chemical composition of *kaštradina* was evident after 35 and 60 days of processing, where the relationships among the three different categories (ewes, wethers, and rams) were similar to those found in fresh meat. Furthermore, the chemical composition of *kaštradina* after 35 days of processing was similar to the composition stated by Dumić [40] in the research of the mentioned “*stelja*” (46.42% to 49.67% of water, 34.66% to 37.12% of proteins, 6.99% to 8.31% of fat, and 7.34% to 9.29% of ash), which differed significantly only in fat content (*kaštradina* from this study contained more fat). That can be explained by the influence of anatomical position (shoulder muscles generally contain more fat than the leg muscles). Furthermore, in comparison with the results of Ganić et al. [44], sheep “*stelja*” (*M. longissimus thoracis et lumborum*) from Bosnia and Hercegovina contained significantly more fat than the *kaštradina* (26.30% in industrial production and 30.92% in craft production) and significantly less proteins (19.90–21.09%), while the values of the other two indicators were similar (40.93–45.75% of water and 5.15–6.83% of ash). The optimal content of intramuscular fat is of great importance for the quality of dry-cured meat products. Many studies have reported its positive effect on sensory characteristics of dry-cured meat products, such as marbling, flavor, juiciness, and tenderness, and the effect on fatty acid composition [45].

Regarding the NaCl content in the *kaštradina*, it can be assumed that the castration procedure affected salt reduction in *kaštradina* after 35 and 60 days of processing (*p* < 0.05), while a significant effect of gender was not found, since the differences between ewes and entire rams were not significant. Significantly lower NaCl content in the *kaštradina* of wethers at the end of processing (60th day) was probably due to higher DM and fat content, which confirmed the high Pearson correlation coefficient (Table 2) between NaCl and DM (*n =* 0.88; *p* < 0.001) and NaCl and F/DM (*n* = 0.89; *p* < 0.001) The results of other authors [1,26,40,44,46] showed significant variability in the NaCl content (3.5% to 11.05%) of similar types of dry-cured mutton that have been traditionally produced in the Balkan region.

### 3.2. Macro- and Microelements of Raw Mutton and “Kaštradina”

This study identified significant differences in the mineral composition of different categories (ewes, wethers, and rams) of raw and processed mutton (Table 3). Regardless of the different relations in mineral content among categories, the lowest contents of macro- and microelements were found in the samples of the wethers’ category, in which a significantly higher content of fat was found, which supports the claim of some authors [47] that fattier meat contains less (up to one-third less) macro- and microelements’ content due to lower inorganic content (ash). There are numerous studies of the mineral composition of lamb [24,48,49,50,51] in contrast to the mineral composition of older categories of mutton [52,53,54], while the data on the mineral composition of dry-cured mutton cannot be found in the available data base.

Table 3 shows that raw mutton of three categories (ewes, wethers, and rams) in the *kaštradina* production differed in K, Na, Zn, and Fe compositions. However, only the content of K in the rams’ category was significantly higher than that found in the other two categories, while the content of Na, Zn, and Fe was significantly lower in the wethers’ category only in comparison to the ewes’ category. Since all macro- and microelements were less present in the wethers’ category of meat, only the difference in K content can be considered as the influence of sex, while the differences in Na, Zn, and Fe contents were obviously due to castration, i.e., increased fat content and reduced content of ash. The higher content of K in the R category (intact adult males) in the present study could be explained by the higher physical activity of rams during mating season. Since the slaughter was performed at the end of the mating season, greater physical activity during matting is implicated in the fatigue process, in which the K^+^ ions are released from contracting muscles in the extracellular compartment [55,56]. Another possible hypothesis is that the higher content of K in R (intact adult male), compared to E (intact adult females) and W category (castrated adult males), is promoted by gender differences. Namely, numerous studies have provided evidence of testosterone vasodilatory activity on the potassium channel-opening effect [57]. Therefore, although serum testosterone levels were not measured in this study, as intact rams (R category) were slaughtered at the end of the mating season, it is very likely that circulating testosterone levels in rams’ serum were high [38,58]. Research of other authors on the sex influence on the mineral composition of meat is different. Thus, many authors report a more significant influence of the geographical breeding region (soil composition and grazing) than the influence of sex [51,59,60]. However, Mioč et al. [50] point out the insignificant influence of sex and breed on the mineral composition of lamb of Istrian sheep and Dalmatian pramenka breed. Concerning the results of this research, a very similar mineral composition was found for lamb of Istrian and Dalmatian pramenka breeds [50] and Spanish Churra breed [48], all bred in the Mediterranean region, so the geographical area of breeding could be the predominant factor of mineral composition of meat (effect of soil composition). However, other factors such as gender and castration are also important.

Changes in the mineral composition of *kaštradina* during processing generally follow the trend established in fresh meat. However, the data in Table 3 confirm the significant effect of processing on all minerals, which is probably due to dehydration and concentration of mineral composition, since Pearson coefficients (*n*) between individual minerals and DM and water are highly positive and negative, respectively (Table 2). However, during the salting phase, a smaller amount of minerals could also be lost through the loss of meat juice. In addition, significant differences in the content of particular macro- and microelements among specific categories in *kaštradina* after 60 days of processing were found in the content of all elements except the Cu content. Additionally, a significantly higher content of Ca was found in the *kaštradina* of E category after 35 and 60 days of processing. Potassium and calcium ions are known to participate in muscle contraction, and calcium ions are necessary for the activity of the endogenous proteolytic enzymes, calpain and calpastatin, that play a key role in postmortem tenderization of skeletal muscles [61]. Therefore, it is to be expected that the higher contents of Ca and K could improve the sensory properties of *kaštradina*, especially its tenderness [61].

## 4. Conclusions

The results of this research indicate a significant influence of sex and castration as well as the dry-curing process on the proximate chemical and mineral compositions of raw mutton and *kaštradina.* A higher intramuscular fat content and a lower content of NaCl found in the *kaštradina* of wethers and ewes could affect its sensory and nutritional properties. Additionally, higher contents of Ca and K found in *kaštradina* of ewes and rams, respectively, could affect its sensory properties, especially tenderness. These differences suggest the mutton of ewes and wethers could be a better-quality raw material in *kaštradina* production than could the meat of rams. However, further research on chemical and sensory properties is needed to provide a more comprehensive picture of its quality.

## Figures and Tables

**Table 1:** Proximate chemical composition of different categories of raw mutton and *kaštradina* in different processing times, %.

Category	Parameters, %								
	Water	DM	Proteins	Fat	Ash	NaCl	P/DM	F/DM	A/DM
1st day of processing									
Ewes	74.28	25.72	20.44 ^a^	5.08 ^a^	1.06 ^a^	-	79.47 ^a^	19.75 ^a^	4.12 ^a^
Wethers	74.42	25.58	17.86 ^b^	7.30 ^b^	0.83 ^b^	-	70.14 ^b^	28.22 ^b^	3.26 ^b^
Rams	75.73	24.27	20.64 ^a^	2.40 ^c^	1.09 ^a^	-	85.20 ^c^	9.70 ^c^	4.50 ^a^
SE	0.46	0.46	0.46	0.69	0.04	-	2.38	2.56	0.15
P	0.596	0.284	0.007	0.038	<0.001	-	0.044	0.009	0.029
35th day of processing									
Ewes	48.52	51.48	33.28 ^a^	10.44 ^a^	8.16 ^a^	5.48 ^a^	64.65 ^a^	20.28 ^a^	15.88 ^a^
Wethers	48.89	51.51	27.57 ^b^	17.31 ^b^	4.85 ^b^	3.70 ^b^	53.64 ^b^	33.71 ^b^	9.50 ^b^
Rams	49.46	50.54	35.25 ^a^	7.79 ^c^	6.73 ^c^	4.99 ^a^	69.77 ^c^	15.38 ^c^	13.32 ^c^
SE	0.84	0.91	0.79	0.79	0.34	0.31	1.23	1.52	0.70
P	0.832	0.407	<0.001	0.021	0.042	0.039	0.025	0.019	0.045
60th day of processing									
Ewes	38.27 ^a^	61.73 ^a^	37.87 ^a^	15.63 ^a^	8.25 ^a^	5.89 ^a^	61.35 ^a^	25.32 ^a^	13.37 ^a^
Wethers	37.68 ^a^	62.32 ^a^	31.38 ^b^	23.48 ^b^	7.68 ^a^	4.37 ^b^	50.42 ^b^	37.56 ^b^	12.37 ^a^
Rams	39.13 ^b^	60.87 ^b^	41.94 ^a^	9.25 ^c^	9.07 ^b^	6.49 ^a^	68.89 ^c^	15.21 ^c^	14.91 ^b^
SE	0.54	0.54	1.22	1.35	0.22	0.29	1.96	2.05	0.40
P	0.028	0.041	0.003	0.008	0.048	0.003	0.037	0.006	0.025
Day of processing									
1st day	74.81 ^a^	25.19 ^a^	19.82 ^a^	5.41 ^a^	0.99 ^a^	-	79.05 ^a^	17.12 ^a^	3.96 ^a^
35th day	48.96 ^b^	51.18 ^b^	33.53 ^b^	10.18 ^b^	6.58 ^b^	4.73	65.59 ^b^	19.89 ^a^	12.90 ^b^
60th day	38.36 ^c^	61.64 ^c^	37.06 ^c^	16.12 ^c^	8.33 ^c^	5.58	60.22 ^b^	26.03 ^b^	13.55 ^b^
SE	0.37	0.39	0.83	1.02	0.20	0.23	1.77	2.05	0.41
P	<0.001	<0.001	0.005	<0.001	<0.001	0.003	<0.001	0.007	<0.001

DM—dry matter; P/DM—proteins per DM; F/DM—fat per DM; A/DM—ash per DM; SE—standard error; a, b, c—different letters within the same column indicate significant differences among sex groups (*p* < 0.05).

**Table 2:** Pearson correlation matrix (n) of the investigated variables.

Variables	Ca	P	Mg	K	Na	Mn	Cu	Zn	Fe	Water	DM	Protein	Fat	Ash	NaCl	P/DM	F/DM
Ca	1	-	-	-	-	-	-	-	-	-	-	-	-	-	-	-	-
P	0.71	1	-	-	-	-	-	-	-	-	-	-	-	-	-	-	-
Mg	0.76	0.97	1	-	-	-	-	-	-	-	-	-	-	-	-	-	-
K	0.59	0.96	0.93	1	-	-	-	-	-	-	-	-	-	-	-	-	-
Na	0.84	0.75	0.81	0.69	1	-	-	-	-	-	-	-	-	-	-	-	-
Mn	0.80	0.77	0.82	0.72	0.81	1	-	-	-	-	-	-	-	-	-	-	-
Cu	0.63	0.65	0.65	0.53	0.52	0.61	1	-	-	-	-	-	-	-	-	-	-
Zn	0.78	0.90	0.93	0.83	0.80	0.80	0.71	1	-	-	-	-	-	-	-	-	-
Fe	0.73	0.70	0.69	0.62	0.64	0.82	0.56	0.69	1	-	-	-	-	-	-	-	-
Water	−0.78	−0.62	−0.70	−0.52	−0.80	−0.68	−0.65	−0.81	−0.53	1	-	-	-	-	-	-	-
DM	0.78	0.62	0.70	0.52	0.80	0.68	0.65	0.81	0.53	−1.00	1	-	-	-	-	-	-
Protein	0.75	0.82	0.83	0.76	0.80	0.76	0.62	0.84	0.65	−0.79	0.79	1	-	-	-	-	-
Fat	0.30	−0.03	0.08	−0.13	0.28	0.14	0.28	0.27	0.04	−0.63	0.63	0.02	1	-	-	-	-
Ash	0.81	0.75	0.81	0.68	0.87	0.78	0.65	0.86	0.61	−0.91	0.91	0.91	0.30	1	-	-	-
NaCl	0.83	0.79	0.83	0.72	0.94	0.80	0.59	0.85	0.63	−0.88	0.88	0.88	0.30	0.94	1	-	-
P/DM	0.06	−0.25	−0.15	−0.31	0.06	−0.06	0.04	0.01	−0.17	−0.38	0.38	−0.25	0.94	0.04	0.06	1	-
F/DM	0.77	0.61	0.71	0.54	0.84	0.70	0.59	0.78	0.51	−0.93	0.93	0.74	0.53	0.95	0.89	0.30	1
A/DM	0.79	0.67	0.74	0.59	0.92	0.73	0.52	0.78	0.54	−0.90	0.90	0.75	0.49	0.89	0.96	0.29	0.93

DM—dry matter; P/DM—proteins per DM; F/DM—fat per DM; A/DM—ash per DM.

**Table 3:** Macro- and microelements of different categories of raw mutton and *kaštradina* in different processing times (mg/kg).

Category	Macro- and Microelements, mg/kg Sample								
	Ca	P	Mg	K	Na	Mn	Cu	Zn	Fe
1st day of processing									
Ewes	42.62	1866.70	198.23	3211.60 ^a^	859.90 ^ab^	0.0781	0.81	30.61 ^a^	30.79 ^a^
Wethers	34.10	1743.70	189.38	3028.20 ^a^	785.90 ^b^	0.0621	0.75	21.87 ^b^	24.70 ^b^
Rams	37.85	1930.70	208.88	3571.70 ^b^	891.60 ^a^	0.0750	0.90	28.17 ^ab^	26.17 ^ab^
SE	3.84	67.98	7.28	79.96	31.91	0.0051	0.07	2.41	1.83
P	0.395	0.098	0.486	0.006	0.049	0.912	0.394	0.031	0.049
35th day of processing									
Ewes	107.62 ^a^	2551.30 ^ab^	282.45 ^ab^	4245.80 ^a^	20,339.20 ^a^	0.1840 ^a^	1.10 ^a^	56.12 ^ab^	46.66 ^a^
Wethers	65.84 ^b^	2114.30 ^a^	238.39 ^a^	3619.90 ^a^	13,594.50 ^b^	0.1119 ^b^	0.93 ^b^	50.62 ^b^	29.17 ^b^
Rams	79.66 ^b^	2985.20 ^b^	323.67 ^b^	5227.00 ^b^	21,157.20 ^a^	0.1516 ^ab^	1.03 ^ab^	66.06 ^a^	41.47 ^a^
SE	7.01	134.60	12.94	251.88	1663.84	0.0142	0.05	3.64	3.48
P	0.043	<0.001	<0.001	0.034	0.017	0.029	0.048	0.019	0.027
60th day of processing									
Ewes	134.41 ^a^	3009.90 ^a^	327.48 ^a^	4774.80 ^a^	22,232.10 ^a^	0.1996 ^a^	1.52	76.34 ^a^	62.43 ^a^
Wethers	99.02 ^b^	2408.10 ^b^	288.61 ^b^	3796.70 ^b^	15,173.50 ^b^	0.1353 ^b^	1.29	72.60 ^a^	35.17 ^b^
Rams	103.33 ^b^	3614.10 ^c^	385.24 ^c^	6290.60 ^c^	20,725.80 ^ab^	0.1857 ^a^	1.36	92.99 ^b^	42.40 ^b^
SE	7.17	104.66	10.11	45.60	1863.26	0.0128	0.08	3.37	3.15
P	0.038	0.009	0.047	<0.001	0.016	0.028	0.472	0.004	<0.001
Day of processing									
1st day	38.19 ^a^	1847.03 ^a^	198.83 ^a^	3270.50 ^a^	845.80 ^a^	0.0717 ^a^	0.82 ^a^	26.88 ^a^	27.22 ^a^
35th day	84.37 ^b^	2550.27 ^b^	281.50 ^b^	4364.23 ^b^	18,363.63 ^b^	0.1492 ^b^	1.02 ^b^	57.60 ^b^	39.10 ^b^
60th day	112.25 ^c^	3010.70 ^c^	333.78 ^c^	4954.03 ^c^	19,377.13 ^b^	0.1735 ^b^	1.39 ^c^	80.64 ^c^	46.67 ^c^
SE	4.29	88.28	8.13	164.72	939.98	0.0077	0.04	2.16	2.20
P	<0.001	0.004	<0.001	0.034	<0.001	<0.001	0.002	<0.001	0.041

SE—standard error; a, b, c—different letters within the same column indicate significant differences among sex groups (*p* < 0.05).

## Data Availability

The data presented in this study are available on request from the corresponding author. The data are not publicly available due to the project agreement.
